# Influence of Humidity on Fatigue Performance of CFRP: A Molecular Simulation

**DOI:** 10.3390/polym13010140

**Published:** 2020-12-31

**Authors:** Bowen Li, Jianzhong Chen, Yong Lv, Li Huang, Xiaoyu Zhang

**Affiliations:** Hubei Key Laboratory of Theory and Application of Advanced Materials Mechanics, School of Science, Wuhan University of Technology, Wuhan 430070, China; soslbw@whut.edu.cn (B.L.); lvyonghl@163.com (Y.L.); wgdHL@163.com (L.H.); zxysandy@126.com (X.Z.)

**Keywords:** fatigue, MD, CFRP, humidity, interface

## Abstract

The study on durability of carbon fiber reinforced plastics (CFRP) in complex environments is critical because of its wide applications. Herein, mechanical behavior of carbon fiber reinforced epoxy composites in the fatigue process were investigated under different humidity via molecular dynamics (MD) simulation method. Transversely isotropic atom based models were established to simulate the structure of CFRP at the atomistic level. Owing to the weak performance in vertical fiber direction, mechanical behavior in a 90° orientation was investigated. Mean stress and energy were both employed to describe the evolution of mechanical performance while mean squared displacement (MSD), radius of gyration (Rg), and free volume were performed to describe the evolution of structural change during the fatigue process. The results show that the humidity led to a weakened interfacial adhesive performance. Free volume became larger under cyclic load, which caused the water molecules to diffuse into the inside of epoxy resin. The distance between the matrix and fiber became larger in the dry system while it reduced because of the diffusion of water molecules in wet system. The rate of performance degradation decreased with the increase in humidity because of poor initial performance at high humidity.

## 1. Introduction 

Owing to their excellent material performance such as extremely high specific modulus, large specific strength, and great conductive properties, carbon fiber reinforced plastics (CFRP) are widely used in the aerospace, civil, construction, power, shipping, and other industries. Therefore, several researchers have proposed that it is necessary to study the fatigue properties of CFRP because of its specific applications and complex fatigue behavior. CFRP can be used in the moving structures such as airplanes and helicopters [1]. When it is used for components in the aircraft engine blades and fuselage, the fatigue behavior should be considered because both these components are subjected to fatigue cyclic loading [2,3]. Compared to metals, the fatigue process of composites is more complex. The fatigue behavior of the composites include fiber breakage, matrix cracking, fiber-matrix debonding, delamination, and effect of shear-induced diffuse damage on the transverse cracks. In general, once there is a damage, it will perpetuates further [4,5,6]. If environmental factors and occasional loading [2] are considered, the damage process is more complicated. Besides, initial damage of composites is often subsurface and non-destructive. Some advanced techniques such as X-ray and Ultrasonic wave [4,6,7] have been employed to investigate the fatigue behavior of FRP. 

Till now, the fatigue performance analysis has primarily been experimental. The general procedures for fatigue tests are similar. The evolution of specimen stiffness is monitored by the response of stress or strain and the microstructural transformation can be observed by checking the sliced samples under electronic microscope [8]. The stress control method and strain control method are generally used to apply the fatigue load [9,10]. Performance degradation is usually assessed by the evolution of strain or stress and mesostructures per cycles. This method is also called residual stiffness method and our research is based on this method. Some other analytical methods such as the residual strength method, energy method, etc. are also used by researchers. The loading amplitude, frequency, and other factors impact the fatigue performance of a material [11,12]. Fatigue performances of the composites investigated via experimental methods listed above are generally studied under laboratory environment where other environmental factors can be ignored. However, composites will in fact be significantly affected by other environmental factors such as humidity, temperature, light, radiation, and so on. Therefore, some researchers have explored the performance degradation under different environments. 

Ma et al. [13] investigated the effect of hygrothermal environment on tension-tension fatigue performance and reliable life of CFRP. Their results show that the degree of damage of the saturated specimens is especially, is more severe than that of the virgin specimens. Bending fatigue performance was tested by Yoshi et al. [14]; the cracks and delamination occurred under 95% relative humidity while the bending stiffness decreased faster than under dry conditions. Also, humidity led to a weaker interfacial performance. Pedro et al. [15] concluded that moisture diffuses into the interface via an adsorption process and accelerates the degradation of adhesive properties. Other researchers [16,17,18] have also unanimously concluded that humidity leads to a faster performance degradation. 

However, unexpected problems arise in our study on influence of humidity on fatigue performance of CFRP in laboratory. The performance of the specimens varied so much that more parallel experiments were required to eliminate data volatility. Also, from our experience with fatigue testing, the occasional power break, machine failure, and other reasons have different degrees of impact on the experimental data. All of these problems lead to a significant increase in the time and money requirements. Otherwise, only structural changes are observed at the micrometer scale and the observation is discontinued. With the development of computer science and theory of molecular simulation, the molecular dynamics (MD) method has been widely used in the field of computational materials science. Materials behavior can be simulated by the partial movement based on Newton’s theory of motion and potential function based on quantum mechanics. 

The MD method is widely used in studying the basic performance of materials, such as tensile properties, shear properties, Young’s modulus [19], glass transition temperature [20,21,22] etc. The MD method is a decent technique for fatigue simulations. It can reflect on the cause of change in material properties at the atomic scale. Besides, due to its characteristics of the time scale, molecular dynamics simulation can be regarded as a natural acceleration method. Some researchers have started to use the MD method to study the long-term performance of materials. Tensile-tensile [23,24] and tensile-compression [25] fatigue of polyethylene were studied by the MD method. All these research works show that the stiffness of materials decreases with cycle numbers. Dihedral energy and van der Waals clearly decrease under fatigue cyclic loading, signifying that the evolution of polymeric chain morphology is an important cause for performance degradation. Besides, both the loading amplitude and frequency influence the rate of degradation of material performance. The results are similar for other polymers such as epoxy resin [26]. Besides, non-linear creep performance of polymer [27], nanocomposites [28] was investigated, and these studies shown creep behaviors in atomic scale. The results discussed above are consistent with those obtained from the experiments. Therefore, this work proves that it is feasible to study fatigue by the MD method. However, study objects of this research work are all isotropic materials and all of them ignore the impact of environment. Especially, for the structural materials, polymeric materials are not employed alone but always fabricated as matrix in composites. 

Herein, we have studied the performance degradation in vertical fiber orientation and explored the influence of humidity on the fatigue performance of composites via MD method. Besides, some of the phenomenon during fatigue are explained at atomic scale. 

## 2. Material and Model Method

Multilayer molecular model is widely used to simulate the composites [29,30,31] and layer model of epoxy/graphene/epoxy was performed to simulate the carbon fiber reinforced polymer [19,30,31]. 

### 2.1. Force Field

The consistent-valence force field (CVFF) was used in the simulation of graphene-epoxy nanocomposites [31]. CVFF consists of bond energy and non-bond potential energy. The equation can be described as:

(1) $$\begin{array}{cc} E_{total} & =E_{bond}+E_{non-bond}\\ & =E_{bond}+E_{angle}+E_{torsion}+E_{out-of-plane}+E_{vdw}+E_{coulomb}\\ & =\sum_{bond}K_{b}{\left(b-b_{0}\right)}^{2}+\sum_{angle}K_{\theta }{\left(\theta -\theta_{0}\right)}^{2}+\sum_{torsion}K_{\phi }{\left(\phi -\phi_{0}\right)}^{2}\\ & +\sum_{improper}K_{\chi }\left[1+\cos\left(n\chi -\chi_{0}\right)\right]+\sum_{vdw}\left[{\left(\frac{A_{ij}}{r_{ij}}\right)}^{12}-{\left(\frac{B_{ij}}{r_{ij}}\right)}^{6}\right]+\sum_{coulomb}\frac{Cq_{i}q_{j}}{\varepsilon r_{ij}} \end{array}$$

*K_b_*, *K_θ_*, *K_Φ_*_,_ and *K_χ_* are the force constants. *b*_0_, *θ*_0_, *Φ*_0_, and *χ*_0_ are the equilibrium bond length, bond angle, torsion angle, and improper angle, while n is the periodicity parameter. *A_ij_* and *B_ij_* are the square root of product between *A_i_* with *A_j_* and *B_i_* with *B_j_*. *C* and *ε* are the energy-conversion constant and permittivity, while *r_ij_* is the distance between the atom *i* and *j* with charges *q_i_* and *q_j_*. 

### 2.2. Materials and Molecular Model

#### 2.2.1. Crosslinked Epoxy Resin and Its Composites

**Figure 1:**
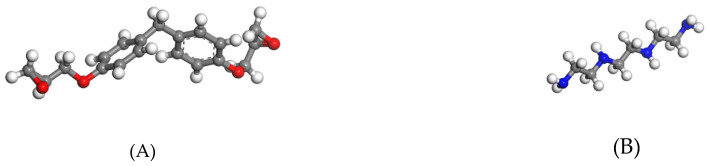
Epoxy resin monomer and curing agent: (**A**) DGEBF; and (**B**) TETA.

Herein, an all-atom model was employed as it is easy to implement. The model employed in Yu’s research [19] is used here. The molecular structures of Bisphenol-F (DGEBF) resin and Triethylenetetramine (TETA) curing agent are shown in Figure 1. 

**Figure 2:**
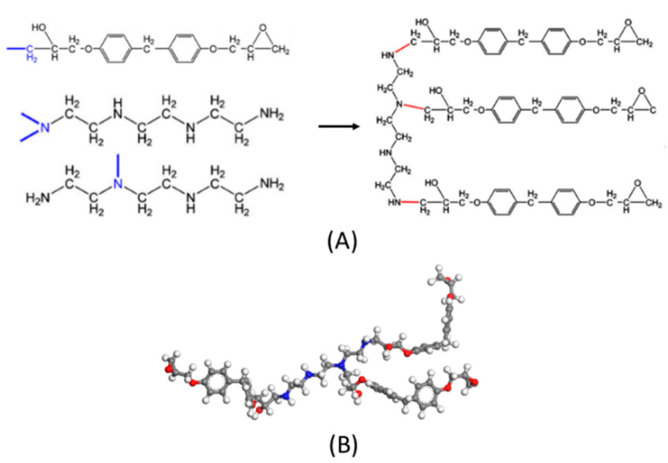
Normal crosslinking procedure and represented molecule. (**A**) crosslinking procedure of epoxy resin; and (**B**) representative molecule [19].

There are two methods to build epoxy resin models. Yu et al. [19] compared these two different methods to establish the epoxy resin molecular model. One is the cross-link producing method, while the other is a representative molecule method. The former method entails placing the epoxy monomers and curing agent monomers into a box to create an amorphous cell, and cross-link script was used to simulate the crosslinking process. The latter method involves fabrication of a representative cross-link unit first, and then, placing the representative units into a box to create an amorphous cell. The results show that the latter method is simpler and closer to the experimental data. Therefore, herein, the latter method was chosen. The normal procedure of crosslinking and the represented molecule can be simply represented as Figure 2. 

**Figure 3:**
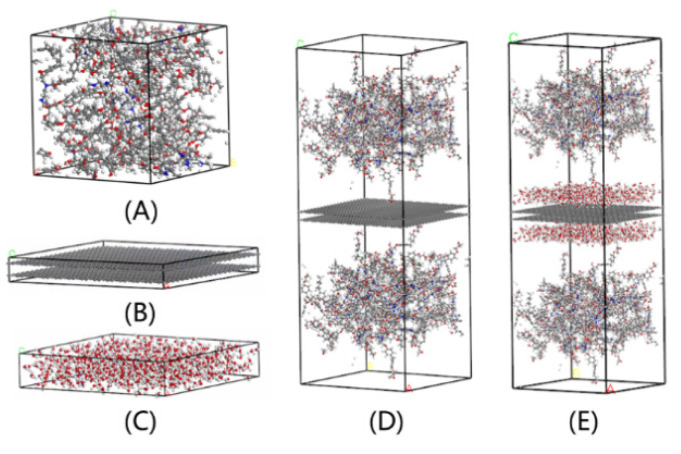
Molecular models of epoxy resin and its composites. (**A**) pure epoxy resin model consists of 15 represented units; (**B**) multi-layers graphene; (**C**) water molecule; (**D**) water layer; (**E**) initial model of epoxy resin enhanced by multi-layers layers graphene; and (**E**) initial enhanced system consisting of water molecules.

An amorphous cell composed of 30 representative molecules can be obtained by using the method chosen above. The crosslinking model with an initial density of 0.8 g/cm^3^ is shown in Figure 3A. The system was built at 298 K and under the periodic boundary conditions. Hereafter, a molecular model of composites with different humidity is required.

The method of constructing graphene reinforced epoxy resin is using the Build Layer module in the commercial software Materials Studio. The build Layer module was used to build multi-layers graphene and epoxy resin as a multi-layered system. 

Polymer-water-graphene-water-polymer model was used to simulate the system with humidity to make the modeling more intuitive and simple. The model for water molecule was TIP3P. Parameters of TIP3P are listed in Table 1.

The moisture content of systems with different humidity are listed in Table 2. Thereafter, the msi2lmp tool was used to obtain the data file that consists of atomic mass, atomic charge, atom coordinates, and topological information of bonds. The data file can be read by LAMMPS, an open source software that is widely used in molecular simulations. 

#### 2.2.2. Equilibrium 

Before performing the strain-control fatigue cyclic loading, an equilibrium system should be first set-up. The Langevin thermostat was applied on the initial structure within NVE ensemble for 2 ns at 298 K to perform Brownian dynamics simulation of a molten polymer. The system was then equilibrated at the NPT ensemble for 2 ns at 298 K with the time step of 0.5 fs. Finally, a stable model was obtained. The NPT ensemble was controlled by the Nose-Hoover thermostat and Nose-Hoover barostat. The time integration of the motion equations was conducted using Velocity-Verlet integration. Final density of equilibrium systems with different humidity are listed in Table 2. 

**Figure 4:**
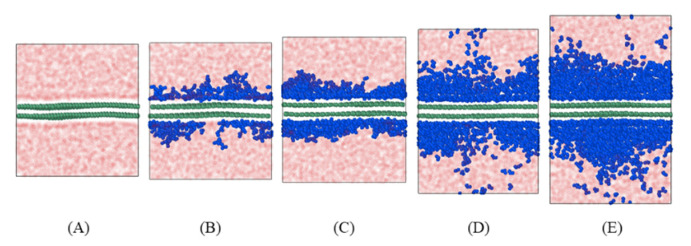
Molecular model of systems in different humidity. (**A**) Model-1, (**B**) Model-2, (**C**) Model-3, (**D**) Model-4, and (**E**) Model-5. Epoxy resin in light red, graphene in green, and water in blue.

Molecular models with different humidity are shown in Figure 4.

## 3. Results and Discussion 

### 3.1. Uniaxial Tensile 

Herein, we primarily investigated the impact of humidity on the fatigue performance and ignored other factors which can be considered in further work. Before applying the fatigue loading, the model was monotonically deformed to the initial strain within the NPT ensembles. The pressure in the unloaded direction was kept at 1 atm. Strain rate was a constant value of 10^−6^/ fs and the time step was 1 fs. Stress calculation method was based on the Virial theory that is widely used in the atomic scale. The whole process was completed using open source software LAMMPS. 

**Figure 5:**
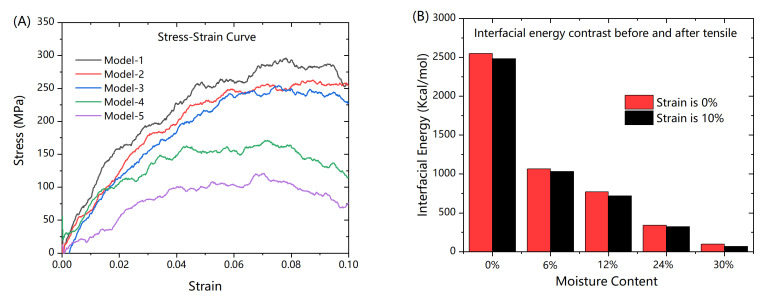
Stress-Strain Curve and evolution of interfacial energy (**A**) Stress-Strain Curve in loading direction, (**B**) Absolute interfacial energy change in loading direction.

The stress-strain curves are shown in Figure 5A. Comparing curves in this figure, Young’s modulus and strength decreased with the increase in humidity. The evolution of interfacial energy, shown in Figure 5B, suggests that the value of absolute initial interface energy decreased with rise of humidity, and slightly amplified during the process of uniaxial tensile. This phenomenon indicates that the presence of water molecules at the interface reduces the bonding efficiency between the epoxy resin and graphene. Besides, stretching increases the distance between the epoxy resin and graphene, and this process leads to a lower interfacial energy.

### 3.2. Tensile-Tensile Fatigue 

Fatigue load controlled by strain was applied to the system and herein, primarily, the fatigue behavior was studied in vertical fiber direction (90°). Fatigue cyclic loading was performed using sinusoidal function in the loading direction, the pressure in the unloaded direction was kept at 1 atm. The loading curve can be expressed as:

(2) $$\varepsilon \left(t\right)=\varepsilon_{0}+Bsin\left(\frac{2\pi }{T}\cdot t\right)$$

where *ε*_0_ is the initial strain, *B* is the amplitude, *t* is the time, and *T* is the period. Herein, *ε*_0_, *B*, and *T* were 5%, 0.5%, and 1000 fs, respectively. Mean stress [13] is widely used to describe the performance of the composites and can be defined as follows:

(3) $$\sigma_{mean}=\frac{\sigma_{min}+\sigma_{max}}{2}$$

where *σ_min_* and *σ_max_* are the minimum stress and maximum stress per cycle. Some researchers have predicted the long-term performance using short-term performance with a certain function. Mallick et al. [32] found that a modified Gerber equation can describe the effect of mean stress on the fatigue strength of composites. Kwofie et al. [33] used an exponential function to predict the fatigue strength and life based on the mean stress. Shang et al. [34] employed the Three-Parameter Power Function to predict the low cycle fatigue life of superalloy and titanium. Herein, power function was used to fit the data points as employed in our previous macroscopic fatigue experiment. A power function has been widely used to describe the relationship between the stiffness and time in the macroscopic fatigue experiment. The power function can be defined as:

(4) $$S=aN^{b}$$

**Figure 6:**
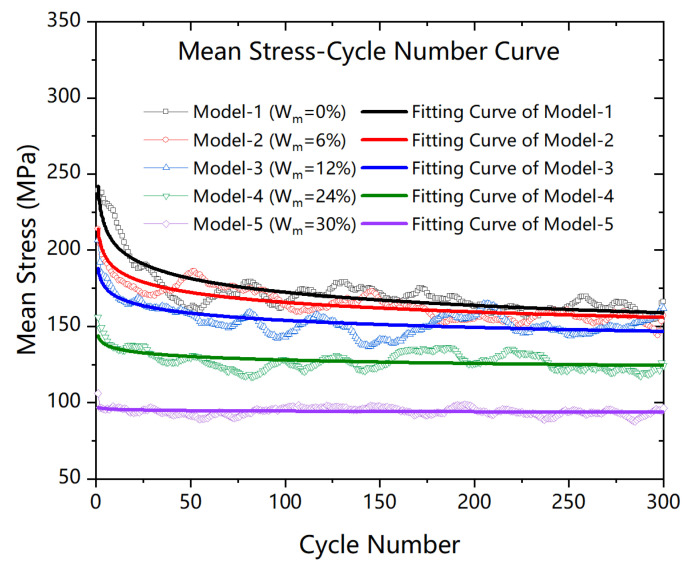
Comparison of mean stress-cycle number curves at different humidity.

(5) lnS=lna+blnN

where *S* is the mean stress per cycle, *N* is the cyclic number, and *a* and *b* are the constant values. Notably, *b* can be regarded as the speed of the performance degradation and a can be regarded as the initial performance. Figure 6 shows the results of the relationship between the mean stress and cycle numbers. 

As shown in Figure 6, with the increase in cycles, mean stress gradually decreased. Comparing mean stress in the first cycle with the increase in humidity, mean stress decreased. This is because the water molecules at the interface reduced the bond efficiency of epoxy resin and graphene. 

**Figure 7:**
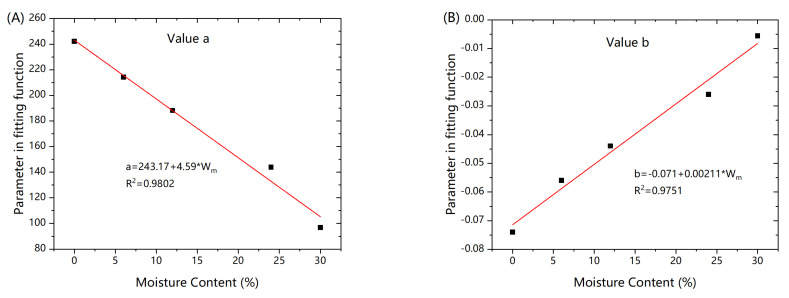
Value a and value b in fitting function. (**A**) comparison of a value under different humidity, (**B**) comparison of b value under different humidity.

Comparing value a and b in each model with increase in humidity in Figure 7, the initial performance and the rate of performance degradation decreased. The performance of composites with high humidity was so poor that no more significant degradation occurs. The evolution of value a and b showed a linear relationship with the change of moisture content. 

### 3.3. Energy Analysis 

For the composites, the total performance and interfacial performance are equally important. Evolution of energy can be used to characterize some microscopic phenomena. In this section, total potential energy and interfacial energy were relatively studied. 

#### 3.3.1. Individual Potential Energy

**Figure 8:**
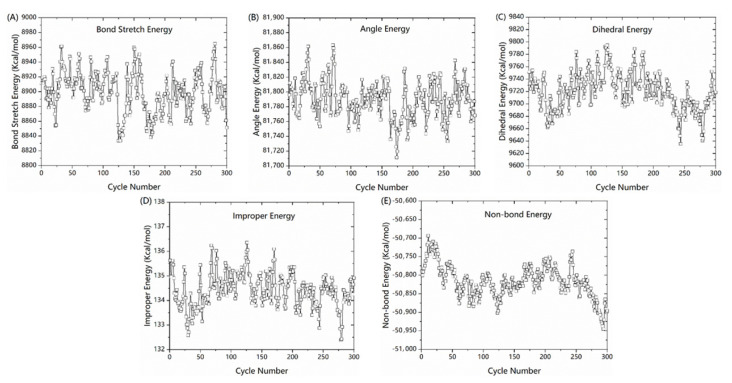
Evolution of individual potential energy in 90° direction. (**A**) bond stretch energy, (**B**) bond angle energy, (**C**) dihedral energy, (**D**) improper energy, and (**E**) non-bond energy.

Total energy includes bond, angle, dihedral, improper, van der Waals, and coulomb energy. Among them, bond, angle, dihedral and improper belong to the bond energy, van der Waals and coulomb belong to non-bond energy. The cause of performance degradation can be analyzed by analyzing the evolution of energy. Herein, systems with different humidity were studied. The results of systems with humidity were almost the same as those of the system without humidity. Therefore, we have only displayed the results of the dry system to avoid figures too jumble. The evolution of individual potential energy is shown in Figure 8.

From Figure 8, non-bond energy clearly decreased while other energies hardly changed. These results indicate that performance degradation in the vertical fiber direction during fatigue is primarily caused by the non-bond interaction. Therefore, the matrix is important in resisting the fatigue load.

#### 3.3.2. Interfacial Energy 

**Figure 9:**
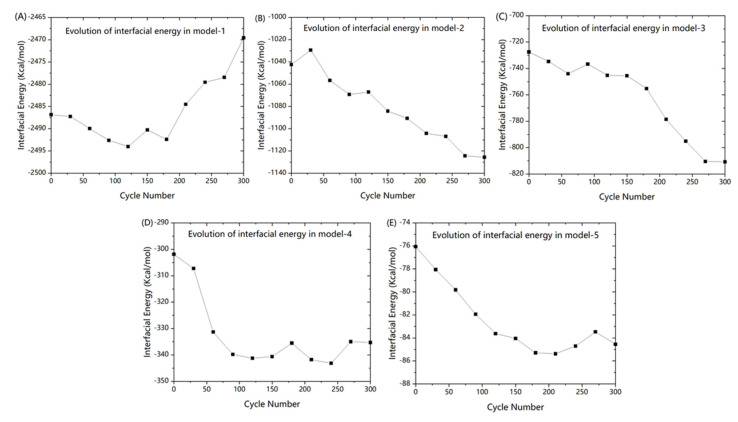
Comparison of energy change in 90° orientation with different humidity. (**A**) model-1; (**B**) model-2; (**C**) model-3; (**D**) model-4; and (**E**) model-5.

Interfacial energy can reflect the change in distance between the epoxy resin and graphene. The evolution of interfacial energy is shown in Figure 9.

As Figure 9A, interfacial energy amplified with cyclic numbers, signifying that the distance between the fiber and matrix became larger. Debonding behavior occurred at the interface. As per Figure 9B–E, it is clear that the interfacial energy decreased with cycle numbers, meaning that the distance between fiber and matrix reduced during fatigue. As the phenomenon seemed to be unexplainable, further analysis was performed on the microstructural change.

### 3.4. Micro-Structure Analysis 

It is critical to investigate the change in structure because macroscopic mechanical response and microscopic energy change are both dependent on the microscopic structural change. In this section, we primarily studied the radius of gyration (Rg), mean-squared displacement (MSD), and free volume fraction (FVF). 

The mean-squared displacement was calculated as the root mean square distance between its centre of mass position of fracture and each monomer position in this fracture:

(6) $$MSD\left(t\right)=\langle {\left|r_{i}\left(t\right)-r_{i}\left(0\right)\right|}^{2}\rangle$$

where *r_i_*(*t*) is the current position of the atom *i* in the group and *r_i_*(0) is the original position of the atom *i* in the group. The x, y, and z components of the mean-squared displacement tensor can be determined using the same formula.

**Figure 10:**
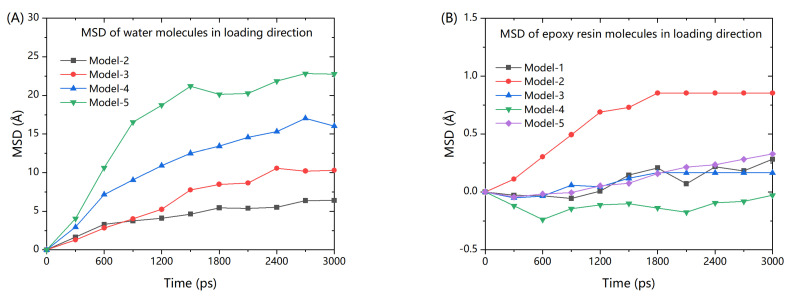
Mean-squared displacement of water molecules and epoxy resin molecules in the loading direction. (**A**) MSD of water molecules; and (**B**) MSD of epoxy resin.

As shown in Figure 10, the MSD of the water molecules about the z-axis (loading direction) clearly increased during the transversal fatigue. The results clearly show that MSD of water was larger than the MSD of epoxy resin. The water molecules gradually diffused into the epoxy resin. More the water molecules at the interface, more was the diffusion.

The radius of gyration was a measure of the size of the group of atoms, and was computed as the square root of the *R_g_*^2^ value in the formula below:

(7) $$R_{g}^{2}=\frac{1}{M}\sum m_{i}{\left(r_{i}-r_{cm}\right)}^{2}$$

where *M* is the total mass of fracture and *r_cm_* is the center of mass position of the fracture. The x, y, and z components of the radius of the gyration tensor can be determined using the same formula. This property indicates the level of deformation. To further clarify the results, the initial value of the radius of gyration was set to 0 and relative mean value of radius of gyration was employed to describe the structural change during the fatigue process. Figure 10 presents the evolution of the relative mean value of radius of gyration during fatigue cyclic loading. 

**Figure 11:**
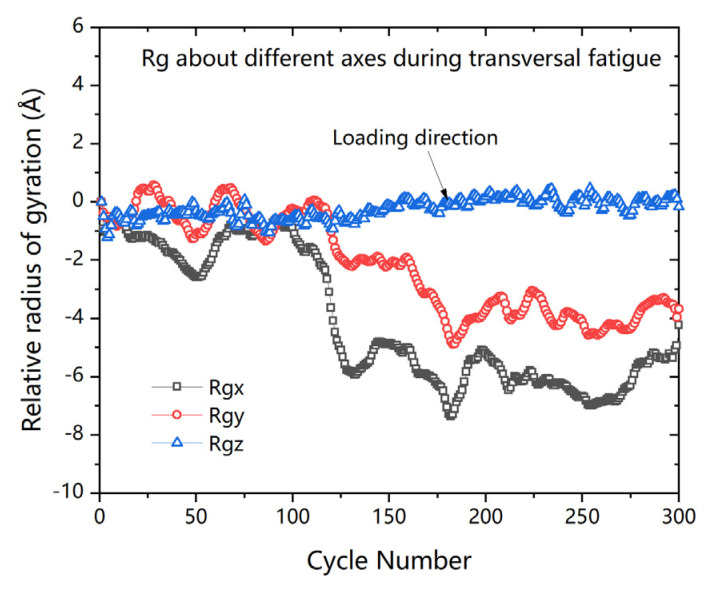
Evolution of the relative radius of gyration during fatigue cyclic loading.

As per Figure 11, the radius of gyration about z-axis (loading direction) increased slightly with number of cycles, while the radius of gyration about other two axes were both decreased during the transversal fatigue. The results of Rg change during fatigue indicate that the molecular fragments tended to align along the loading direction during the fatigue cyclic loading. It can be considered as one of the reasons for performance degradation. 

Free volume fraction which reflects the porosity in a molecular system was explored to study the diffusion conditions of water molecules. A molecular model consisting of pure epoxy resin was established and same fatigue cyclic loading method was applied to the pure epoxy system as before. The number of representative units here was same as it was in the composites molecular model above. The free volume fraction was calculated using Multiwfn, an open source software package. 

**Figure 12:**
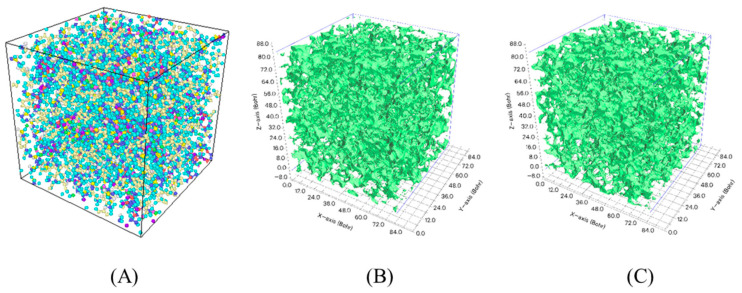
Free volume calculation (**A**) Molecular model; (**B**) Isosurface of primitive free region before fatigue; and (**C**) The isosurface of primitive free region after fatigue Isosurface in green and the free volume in white.

Figure 12 presents the isosurface of the primitive free region. The free region seemed to have hardly changed in the figure. Therefore, it is difficult to visually compare the structural differences between these two models. However, the calculation results displayed that the free volume fraction is up to 10.84% from 10.67%. Greater free volume provides favorable conditions for the diffusion of water molecules.

## 4. Conclusions

Molecular dynamics simulations were used to assess the effect of humidity on the fatigue performance of composites. Four molecular models of composites with different moisture contents were formulated, and for comparison, a molecular model without moisture content was also established. Mean stress was calculated to describe the change in mechanical performance. Interfacial energy was used to describe the interfacial adhesive performance. Mean square displacement and radius of gyration were both used to explain the microstructural change in the process of fatigue. Main conclusions are as follows:

(1) Presence of water in the polymer-graphene interface weakened the stiffness and interfacial adhesive performance in the vertical fiber direction. Higher humidity led to a weakened stiffness and adhesive performance.
(2) During the process of transversal fatigue, molecular fragments tended to align along the loading direction leading to performance degradation. With regards to microscopic energy, this phenomenon is primarily reflected by the decline of van der Waals, while in terms of macroscopic performance, it is mainly reflected by the decrease in mean stress.
(3) During the process of fatigue, interfacial energy of dry system decreased with the cycle numbers. This phenomenon indicates that the distance became larger. However, different results and increase in interfacial energy were observed in the wet system. To understand this phenomenon, another molecular model consisting of pure epoxy resin was established and the free volume of the model before fatigue and after fatigue were relatively calculated. Free volume amplified slightly after fatigue. Increase in the free volume in epoxy resin made it easy for the water molecules to diffuse into the inside of epoxy resin. The diffusion of water molecules reduced the water layer in the polymer-graphene. The thinner water layer led to a stronger interfacial adhesion properties. This conclusion can be confirmed by the evolution of interfacial energy and the mean squared displacement of the epoxy resin and water molecules during fatigue. Besides, interfacial energy of the dry system decreased with the cycle numbers. This phenomenon indicates that the distance became larger.
(4) Higher humidity led to a slower rate of performance degradation during fatigue as the performance degraded fast in first several cycles and less prominently in the last several cycles. The performance of composites with high humidity was so poor that no more significant degradation occurred. However, performance continues to decrease because molecular arrangement is the main cause of the performance degradation, even if the interfacial performance improved slightly. In addition, initial performance and rate of performance degradation show a linear relationship with the change of moisture content.

This research can provide reference for prospective anti-fatigue design for composites in humidity environment.

## Figures and Tables

**Table 1:** Simulation parameters of TIP3P.

Type	LJ Epsilon/Sigma of O	LJ Epsilon/Sigma of H	K/r0 of OH Bond	K/theta of HOH Angle
Parameters	0.102/3.188	0.0/0.0	450/0.9572	55/104.52

**Table 2:** Final density of systems with different humidity.

	Model-1	Model-2	Model-3	Model-4	Model-5
Moisture content (%)	0	6%	12%	24%	30%
Final density (g/cm^3^)	1.316	1.298	1.276	1.209	1.150

## Data Availability

Data available on request due to restrictions eg privacy or ethical. The data presented in this study are available on request from the corresponding author. The data are not publicly availa-ble the data are also forms part of an ongoing study.
